# Longitudinal association between adolescent work values and mental health and well-being in adulthood: a 23-year prospective cohort study

**DOI:** 10.1038/s41598-020-70507-y

**Published:** 2020-08-11

**Authors:** Maiko Fukasawa, Kazuhiro Watanabe, Daisuke Nishi, Norito Kawakami

**Affiliations:** Department of Mental Health, Graduate School of Medicine, The University of Tokyo, 7-3-1 Hongo Bunkyo-ku, Tokyo, 113-0033 Japan

**Keywords:** Risk factors, Signs and symptoms

## Abstract

Personal values developed in adolescence may influence mental health and well-being over the life course. Using data from 684 respondents in the Youth Development Study (61.9% of respondents in the baseline survey), we explored the effects of work values at ages 14–15 on positive and negative emotions, as well as psychological resources (self-esteem and mastery), at ages 37–38. We adjusted for socioeconomic status and the baseline scores of these outcomes using linear regression analyses. Having the work value of getting ahead in adolescence was positively associated with self-esteem in adulthood. Work values in adolescence did not predict positive emotions, negative emotions, or mastery in adulthood. Valuing one’s growth in adolescence may help develop self-esteem in adulthood.

## Introduction

Personal values are defined as broad, trans-situational, desirable goals that serve as guiding principles in people’s lives^1^. A well-known theory of personal values proposed by Schwartz^2^ classified personal values into 10 types. These types of personal values have been associated with mental health and psychological well-being. For example, the values of universalism, benevolence, achievement, hedonism, self-direction, and stimulation have been positively correlated with psychological well-being^3–5^. Personal values have also been associated with psychological resources underlying mental health and well-being, such as self-esteem and mastery^4,6,7^.

Personal values are considered to be developed through the psychological processes in which adolescents acquire the ability to control the conflict between learned values and actual behaviour^8^ and are highly stable across time^9^. Thus, personal values acquired in adolescence may have an influence on health and well-being over the life course. Unfortunately, there is limited evidence supporting this hypothesis. For example, cross-sectional studies based on respondents’ retrospective recall reported that personal values in adolescence were associated with lifetime suicidal ideation, as well as suicidal ideation and psychological distress in adulthood, in a community population^10,11^. A cross-sectional study conducted among working populations reported that personal values in adolescence were associated with mental health and well-being in adulthood^12^. However, to date, we are unaware of any longitudinal study that has been conducted to explore the association between personal values in adolescence and health and well-being in adulthood.

Work values are the goals or rewards people seek through their work^13^, and are seen as expressions of more general personal values in the context of a work setting^14,15^. Work values are often studied in two dimensions: intrinsic (e.g. interesting work, meaningfulness, learning opportunities, etc.) and extrinsic rewards (e.g. pay, security, prestige, opportunities for advancement, etc.)^15–20^. Work values have been associated with the mental health and well-being of workers^15,21–24^, as well as employment^25^, work satisfaction^15,26,27^, occupational choice^28^, person-job fit^25^, and occupational attainment^18,19,29,30^. A limited number of studies have examined the association between the work values of adolescents before they enter the workforce and work-related outcomes in adulthood, and the findings indicated that work values held in high school predicted further educational attainment^16,29^ and work rewards gained as young adults^18,29,30^. However, a longitudinal link between work values in adolescence and mental health and well-being in adulthood has not been explored. While work values are a domain-specific component of personal values, a longitudinal study investigating the association of work values in adolescence with mental health and well-being in adulthood may be useful in understanding the life-course impact of personal values on mental health and well-being.

In this study, we aimed to examine the effects of work values in adolescence on mental health and well-being, as well as on psychological resources (e.g. self-esteem and mastery), in adulthood, using data from a 23-year longitudinal study that tracked individuals from adolescence to young adulthood. In exploring the effects of work values on mental health and well-being, we examined their effects on negative and positive emotions separately, due to the different functions of positive emotions from negative emotions proposed in the broaden-and-build theory. This theory posits that positive emotions broaden people’s momentary thought-action repertoire and build their enduring personal resources, which enhance their well-being, while negative emotions narrow their momentary thought-action repertoire to promote quick and decisive action in life-threatening situations^31^. In analysis, we further adjusted for the baseline score of these outcomes to more strictly explore a causal effect of work values in adolescence on these adulthood outcomes.

## Results

We analysed data from the Youth Development Study (YDS)^32^. We used the data obtained in the baseline survey conducted in 1988 (Time 1) and the latest survey conducted in 2011 (Time 2). Among 1,105 respondents in the baseline survey, 688 participated in the Time 2 survey. Excluding four participants who did not respond to any of the 10 items on work values at Time 1, we used the data of 684 respondents (61.9% of the Time 1 respondents). Compared to the respondents used in the present study, individuals who were lost to follow-up were more likely to be men, non-white, and have a low household income. As for work values, they generally attached less importance on employment security, getting ahead, working with people rather than things, and making one’s own decisions. Additionally, their mastery at Time 1 was lower than that of the respondents used in the present study (see Supplemental Table S1).

Sociodemographic characteristics of the respondents used in the present study are reported in Table 1. Of those respondents, 57.8% were women and 72.8% were white. In Time 2, when the respondents were 37–38 years old, 35.4% had graduated from university or higher, 81.1% were employed, and 71.9% were married or cohabiting. Mean scores of work values, mental health and well-being, and psychological resources are shown in Table 2. Correlations between work values at Time 1 are presented in Supplemental Table S2. We calculated these descriptive statistics using the respondents without missing values on respective variables. For examining associations among these variables as reported in Tables 3, 4, 5, 6, 7, 8, 9 and 10, we handled these missing values using multiple imputation. We reported the results of comparable analyses conducted among only the respondents without missing values in Supplemental Tables S3–S10.

**Table 1:** Socio-economic status of the subjects (N = 684).

	N	%
**Time 1**		
Sex		
Men	278	40.6
Women	395	57.8
Missing	11	1.6
Race		
White	498	72.8
Black	45	6.6
Hispanic	30	4.4
Southeast Asian	51	7.5
Other Asian	7	1.0
Native American	3	0.4
Mixed race	31	4.5
Others	5	0.7
Missing	14	2.1
Total household income in 1987 (parent's report)		
Under $5,000	17	2.5
$5,000–9,999	60	8.8
$10,000–14,999	48	7.0
$15,000–19,999	54	7.9
$20,000–29,999	116	17.0
$30,000–39,999	105	15.4
$40,000–49,999	104	15.2
$50,000–59,999	72	10.5
$60,000–69,999	35	5.1
$70,000–79,999	19	2.8
$80,000–89,999	8	1.2
$90,000–99,999	8	1.2
$10,000 or more	5	0.7
Missing	33	4.8
**Time 2**		
Educational attainment		
High school diploma	126	18.4
Some college	316	46.2
Bachelor's degree or higher	242	35.4
Employment		
Currently employed	555	81.1
No	126	18.4
Missing	3	0.4
Marital status		
Currently married or cohabiting in an intimate relationship	492	71.9
No	187	27.3
Missing	5	0.7
Total household income in 2010		
Less than $50,000	191	27.9
$50,000–$90,000	222	32.5
More than $90,000	207	30.3
Missing	64	9.4

**Table 2:** Work values and mental health and well-being and psychological resources at Time 1 and Time 2.

		Time 1			Time 2		
		N	Mean	SD	N	Mean	SD
**Work values (range: 1–4)**							
1	Good pay	684	3.7	0.5	682	3.2	0.7
2	A steady job, with little chance of being laid off	683	3.6	0.7	681	3.3	0.7
3	Good chances of getting ahead	681	3.6	0.6	679	2.9	0.8
4	A chance to be helpful to others or useful to society	684	3.0	0.8	678	2.8	0.8
5	A chance to work with people rather than things	684	3.0	0.9	682	2.6	0.9
6	A chance to make my own decisions	684	3.2	0.7	681	2.9	0.7
7	A job where I have a lot of responsibility	678	3.0	0.8	680	2.5	0.8
8	A job that uses my skills and abilities	684	3.5	0.7	682	3.2	0.7
9	A job that people regard highly	680	3.0	0.9	682	2.3	0.9
10	A chance to learn a lot of new things	684	3.4	0.7	680	2.8	0.8
	Extrinsic orientations (mean score of 4 items; #1 #2 #3 #9)	677	3.5	0.5	678	2.9	0.6
	Intrinsic orientations (mean score of 6 items; #4 #5 #6 #7 #8 #10)	678	3.2	0.5	674	2.8	0.6
**Mental health and well-being and psychological resources**							
Positive emotions (range 4–20)		613	13.5	2.5	682	13.2	2.6
Negative emotions (range 5–25)		605	12.9	3.8	680	11.3	3.8
Self-esteem (range 7–28)		598	20.0	3.4	682	22.2	3.7
Mastery (range 7–28)		605	20.2	3.0	675	22.1	3.3

*SD* standard deviation.

Tables 3 and 4 show the results of the linear regression analyses of positive emotions. In bivariate analyses, higher intrinsic orientations at Time 1 were related with higher positive emotions at Time 2. However, when both extrinsic and intrinsic orientations were included in a model simultaneously, the correlation coefficient became non-significant (Model 1). The results of a comparable analysis of negative emotions are shown in Tables 5 and 6. None of work values or orientations at Time 1 were significantly associated with negative emotions at Time 2.

**Table 3:** The association between work values at Time 1 with positive emotions at Time 2 applying linear regression analysis (N = 684).

		Bivariate associations				Multivariate analyses											
		Coef	SE	β	p	Model 1^1)^				Model 2^2)^				Model 3^3)^			
						Coef	SE	β	p	Coef	SE	β	p	Coef	SE	β	p
**Work values at Time 1**																	
1	Good pay	− 0.07	0.18	− 0.01	0.696	− 0.19	0.21	− 0.04	0.351	− 0.19	0.21	− 0.04	0.371	− 0.16	0.20	− 0.03	0.421
2	A steady job, with little chance of being laid off	− 0.07	0.14	− 0.02	0.637	− 0.21	0.17	− 0.06	0.205	− 0.23	0.17	− 0.06	0.169	− 0.22	0.17	− 0.06	0.198
3	Good chances of getting ahead	0.27	0.16	0.06	0.097	0.27	0.20	0.06	0.170	0.28	0.20	0.07	0.158	0.27	0.19	0.06	0.159
4	A chance to be helpful to others or useful to society	0.18	0.12	0.06	0.123	0.05	0.15	0.02	0.730	0.09	0.16	0.03	0.561	0.07	0.15	0.02	0.643
5	A chance to work with people rather than things	0.16	0.11	0.05	0.169	0.02	0.14	0.01	0.882	0.03	0.15	0.01	0.844	− 0.01	0.14	0.00	0.963
6	A chance to make my own decisions	0.11	0.13	0.03	0.396	− 0.05	0.16	− 0.01	0.749	− 0.07	0.16	− 0.02	0.689	− 0.09	0.16	− 0.03	0.580
7	A job where I have a lot of responsibility	0.18	0.12	0.06	0.145	0.07	0.15	0.02	0.629	0.08	0.15	0.03	0.597	0.07	0.15	0.02	0.647
8	A job that uses my skills and abilities	0.29	0.15	0.07	0.052	0.21	0.18	0.05	0.255	0.19	0.19	0.05	0.305	0.14	0.18	0.03	0.459
9	A job that people regard highly	0.17	0.11	0.06	0.111	0.12	0.12	0.04	0.325	0.13	0.12	0.05	0.296	0.13	0.12	0.05	0.267
10	A chance to learn a lot of new things	0.20	0.14	0.05	0.163	0.02	0.16	0.01	0.888	0.03	0.16	0.01	0.866	− 0.03	0.16	− 0.01	0.855

*Coef*. regression coefficient, *SE* standard error, *β* standardized regression coefficient.

^1)^Ten work values were entered into a model simultaneously; VIF = 1.25–1.62; R-squared = 0.0149251; adjusted R-squared = 0.0002881; F = 1.01 (p = 0.4364).

^2)^Adjusted for sex, race, and household income at Time 1; VIF = 1.08–1.70; R-squared = 0.0183993; adjusted R-squared = −0.0006467; F = 0.94 (p = 0.5094).

^3)^Adjusted for Model 2 + positive emotions at Time 1; VIF = 1.05–1.70; R-squared = 0.0506491; adjusted R-squared = 0.0307823; F = 2.50 (p = 0.0018).

**Table 4:** The association between work orientations at Time 1 with positive emotions at Time 2 applying linear regression analysis (N = 684).

	Bivariate associations				Multivariate analyses											
	Coef	SE	β	p	Model 1^1)^				Model 2^2)^				Model 3^3)^			
					Coef	SE	β	p	Coef	SE	β	p	Coef	SE	β	p
**Work orientations at Time 1**																
Extrinsic orientations (mean score of 4 items; #1 #2 #3 #9)	0.18	0.21	0.03	0.390	− 0.01	0.23	0.00	0.965	− 0.02	0.23	0.00	0.918	0.01	0.23	0.00	0.959
Intrinsic orientations (mean score of 6 items; #4 #5 #6 #7 #8 #10)	0.38	0.19	0.08	0.043	0.39	0.21	0.08	0.068	0.43	0.22	0.09	0.052	0.23	0.22	0.05	0.301

*Coef*. regression coefficient, *SE* standard error, *β* standardized regression coefficient.

^1)^Two orientations were entered into a model simultaneously; VIF = 1.25–1.25; R-squared = 0.0061702; adjusted R-squared = 0.0032515; F = 2.05 (p = 0.1301).

^2)^Adjusted for sex, race, and household income at Time 1; VIF = 1.06–1.31; R-squared = 0.0089353; adjusted R-squared = 0.0016266; F = 1.16 (p = 0.3274).

^3)^Adjusted for Model 2 + positive emotions at Time 1; VIF = 1.05–1.36; R-squared = 0.0420468; adjusted R-squared = 0.0335568; F = 4.80 (p = 0.0001).

**Table 5:** The association between work values at Time 1 with negative emotions at Time 2 applying linear regression analysis (N = 684).

		Bivariate associations				Multivariate analyses											
		Coef	SE	β	p	Model 1^1)^				Model 2^2)^				Model 3^3)^			
						Coef	SE	β	p	Coef	SE	β	p	Coef	SE	β	p
**Work values at Time 1**																	
1	Good pay	0.42	0.27	0.06	0.117	0.38	0.30	0.05	0.212	0.39	0.30	0.06	0.200	0.32	0.30	0.05	0.281
2	A steady job, with little chance of being laid off	0.30	0.21	0.05	0.153	0.31	0.25	0.06	0.216	0.32	0.25	0.06	0.197	0.36	0.25	0.07	0.148
3	Good chances of getting ahead	− 0.09	0.24	− 0.02	0.697	− 0.36	0.29	− 0.06	0.216	− 0.39	0.29	− 0.06	0.182	− 0.40	0.29	− 0.06	0.169
4	A chance to be helpful to others or useful to society	− 0.03	0.18	− 0.01	0.854	0.03	0.22	0.01	0.886	− 0.03	0.23	− 0.01	0.884	− 0.06	0.23	− 0.01	0.786
5	A chance to work with people rather than things	− 0.05	0.17	− 0.01	0.787	− 0.05	0.21	− 0.01	0.809	− 0.09	0.21	− 0.02	0.676	− 0.08	0.21	− 0.02	0.706
6	A chance to make my own decisions	0.06	0.20	0.01	0.760	0.01	0.24	0.00	0.982	0.02	0.24	0.00	0.921	0.04	0.24	0.01	0.866
7	A job where I have a lot of responsibility	0.08	0.18	0.02	0.649	0.08	0.22	0.02	0.714	0.06	0.22	0.01	0.772	0.05	0.22	0.01	0.832
8	A job that uses my skills and abilities	− 0.07	0.22	− 0.01	0.751	− 0.15	0.27	− 0.03	0.582	− 0.12	0.27	− 0.02	0.659	− 0.06	0.27	− 0.01	0.813
9	A job that people regard highly	0.18	0.16	0.04	0.273	0.18	0.18	0.04	0.309	0.18	0.18	0.04	0.325	0.11	0.18	0.03	0.538
10	A chance to learn a lot of new things	− 0.13	0.21	− 0.02	0.529	− 0.16	0.24	− 0.03	0.508	− 0.16	0.24	− 0.03	0.502	− 0.10	0.24	− 0.02	0.689

*Coef*. regression coefficient, *SE* standard error, *β* standardized regression coefficient.

^1)^Ten work values were entered into a model simultaneously; VIF = 1.25–1.62; R-squared = 0.0106281; adjusted R-squared = − 0.0040728; F = 0.71 (p = 0.7163).

^2)^Adjusted for sex, race, and household income at Time 1; VIF = 1.08–1.70; R-squared = 0.0172894; adjusted R-squared = − 0.0017781; F = 0.88 (p = 0.5711).

^3)^Adjusted for Model 2 + negative emotions at Time 1; VIF = 1.05–1.70; R-squared = 0.0441872; adjusted R-squared = 0.0241851; F = 2.16 (p = 0.0079).

**Table 6:** The association between work orientations at Time 1 with negative emotions at Time 2 applying linear regression analysis (N = 684).

	Bivariate associations				Multivariate analyses											
	Coef	SE	β	p	Model 1^1)^				Model 2^2)`^				Model 3^3)^			
					Coef	SE	β	p	Coef	SE	β	p	Coef	SE	β	p
**Work orientations at Time 1**																
Extrinsic orientations (mean score of 4 items; #1 #2 #3 #9)	0.40	0.30	0.05	0.189	0.51	0.34	0.06	0.135	0.54	0.34	0.07	0.116	0.45	0.34	0.06	0.183
Intrinsic orientations (mean score of 6 items; #4 #5 #6 #7 #8 #10)	− 0.02	0.28	0.00	0.936	− 0.23	0.31	− 0.03	0.465	− 0.34	0.32	− 0.05	0.290	− 0.28	0.32	− 0.04	0.381

*Coef*. regression coefficient, *SE* standard error, *β* standardized regression coefficient.

^1)^Two orientations were entered into a model simultaneously; VIF = 1.25–1.25; R-squared = 0.0034327; adjusted R-squared = 0.0005059; F = 1.12 (p = 0.3259).

^2)^Adjusted for sex, race, and household income at Time 1; VIF = 1.06–1.31; R-squared = 0.0095641; adjusted R-squared = 0.00226; F = 1.25 (p = 0.2842).

^3)^Adjusted for Model 2 + negative emotions at Time 1; VIF = 1.04–1.32; R-squared = 0.0371918; adjusted R-squared = 0.0286588; F = 4.22 (p = 0.0004).

The results of a comparable analysis of self-esteem are shown in Tables 7 and 8. In bivariate analyses, those who valued chances of getting ahead, using one’s skills and abilities, and chances to learn new things at Time 1 were more likely to have higher self-esteem at Time 2. Higher intrinsic orientations at Time 1 were also related with higher self-esteem at Time 2. The associations of the work value of getting ahead and intrinsic orientations with self-esteem remained significant after adjusting for sex, race, and household income at Time 1 (Model 2). However, when self-esteem at Time 1 was added, only the association related to the value of getting ahead remained significant (Model 3).

**Table 7:** The association between work values at Time 1 with self-esteem at Time 2 applying linear regression analysis (N = 684).

		Bivariate associations				Multivariate analyses											
		Coef	SE	β	p	Model 1^1)^				Model 2^2)^				Model 3^3)^			
						Coef	SE	β	p	Coef	SE	β	p	Coef	SE	β	p
**Work values at Time 1**																	
1	Good pay	0.28	0.26	0.04	0.288	0.07	0.29	0.01	0.818	0.07	0.29	0.01	0.819	0.20	0.28	0.03	0.490
2	A steady job, with little chance of being laid off	0.06	0.20	0.01	0.758	− 0.30	0.24	− 0.06	0.203	− 0.33	0.24	− 0.06	0.166	− 0.29	0.23	− 0.06	0.211
3	Good chances of getting ahead	0.64	0.23	0.10	0.006	0.65	0.28	0.11	0.021	0.67	0.28	0.11	0.016	0.64	0.27	0.11	0.017
4	A chance to be helpful to others or useful to society	− 0.01	0.17	0.00	0.964	− 0.41	0.22	− 0.09	0.062	− 0.33	0.22	− 0.07	0.135	− 0.35	0.21	− 0.08	0.098
5	A chance to work with people rather than things	0.23	0.16	0.06	0.148	0.22	0.20	0.05	0.283	0.25	0.21	0.06	0.227	0.17	0.20	0.04	0.399
6	A chance to make my own decisions	0.34	0.19	0.07	0.072	0.10	0.23	0.02	0.659	0.08	0.23	0.02	0.738	0.12	0.22	0.02	0.595
7	A job where I have a lot of responsibility	0.28	0.17	0.06	0.110	0.12	0.21	0.03	0.572	0.14	0.21	0.03	0.519	0.06	0.20	0.01	0.761
8	A job that uses my skills and abilities	0.55	0.21	0.10	0.010	0.34	0.26	0.06	0.190	0.30	0.26	0.05	0.247	0.25	0.25	0.04	0.322
9	A job that people regard highly	0.07	0.16	0.02	0.632	− 0.16	0.17	− 0.04	0.357	− 0.15	0.17	− 0.04	0.390	− 0.11	0.17	− 0.03	0.513
10	A chance to learn a lot of new things	0.43	0.20	0.08	0.034	0.22	0.23	0.04	0.338	0.23	0.23	0.04	0.325	0.13	0.22	0.02	0.573

*Coef*. regression coefficient, *SE* standard error, *β* standardized regression coefficient.

^1)^Ten work values were entered into a model simultaneously; VIF = 1.25–1.62; R-squared = 0.0257019; adjusted R-squared = 0.0112249; F = 1.77 (p = 0.0629).

^2)^Adjusted for sex, race, and household income at Time 1; VIF = 1.08–1.70; R-squared = 0.0334835; adjusted R-squared = 0.0147302; F = 1.76 (p = 0.0464).

^3)^Adjusted for Model 2 + self-esteem at Time 1; VIF = 1.08–1.70; R-squared = 0.1117335; adjusted R-squared = 0.0931449; F = 5.91 (p < 0.0001).

**Table 8:** The association between work orientations at Time 1 with self-esteem at Time 2 applying linear regression analysis (N = 684).

	Bivariate associations				Multivariate analyses											
	Coef	SE	β	p	Model 1^1)^				Model 2^2)^				Model 3^3)^			
					Coef	SE	β	p	Coef	SE	β	p	Coef	SE	β	p
**Work orientations at Time 1**																
Extrinsic orientations (mean score of 4 items; #1 #2 #3 #9)	0.45	0.29	0.06	0.131	0.20	0.33	0.03	0.536	0.17	0.33	0.02	0.615	0.36	0.32	0.05	0.263
Intrinsic orientations (mean score of 6 items; #4 #5 #6 #7 #8 #10)	0.58	0.27	0.08	0.032	0.50	0.30	0.07	0.099	0.63	0.31	0.09	0.043	0.31	0.30	0.04	0.304

*Coef*. regression coefficient, *SE* standard error, *β* standardized regression coefficient.

^1)^Two orientations were entered into a model simultaneously; VIF = 1.25–1.25; R-squared = 0.0074112; adjusted R-squared = 0.0044961; F = 2.51 (p = 0.0820).

^2)^Adjusted for sex, race, and household income at Time 1; VIF = 1.06–1.31; R-squared = 0.0169571; adjusted R-squared = 0.0097075; F = 2.25 (p = 0.0478).

^3)^Adjusted for Model 2 + self-esteem at Time 1; VIF = 1.07–1.34; R-squared = 0.0973875; adjusted R-squared = 0.0893879; F = 11.81 (p < 0.0001).

The results of a comparable analysis of mastery are shown in Tables 9 and 10. In bivariate analyses, those who placed a higher value on employment security, having responsibilities, and using one’s skills and abilities at Time 1 were more likely to have higher mastery at Time 2. Higher intrinsic orientations at Time 1 were also related with higher mastery at Time 2. However, these significant associations did not remain after including the other work values or work orientations simultaneously in a model (Model 1).

**Table 9:** The association between work values at Time 1 with mastery at Time 2 applying linear regression analysis (N = 684).

		Bivariate associations				Multivariate analyses											
		Coef	SE	β	p	Model 1^1)^				Model 2^2)^				Model 3^3)^			
						Coef	SE	β	p	Coef	SE	β	p	Coef	SE	β	p
**Work values at Time 1**																	
1	Good pay	0.29	0.24	0.05	0.216	0.05	0.26	0.01	0.862	0.07	0.27	0.01	0.793	0.18	0.26	0.03	0.499
2	A steady job, with little chance of being laid off	0.38	0.18	0.08	0.036	0.22	0.22	0.05	0.306	0.18	0.22	0.04	0.413	0.11	0.21	0.02	0.601
3	Good chances of getting ahead	0.34	0.21	0.06	0.112	0.10	0.25	0.02	0.695	0.11	0.25	0.02	0.652	0.04	0.25	0.01	0.865
4	A chance to be helpful to others or useful to society	0.06	0.15	0.02	0.684	− 0.21	0.20	− 0.05	0.294	− 0.16	0.20	− 0.04	0.438	− 0.17	0.20	− 0.04	0.378
5	A chance to work with people rather than things	0.22	0.15	0.06	0.135	0.22	0.19	0.06	0.237	0.22	0.19	0.06	0.243	0.23	0.18	0.06	0.213
6	A chance to make my own decisions	0.33	0.17	0.07	0.057	0.09	0.21	0.02	0.653	0.07	0.21	0.02	0.746	0.02	0.20	0.01	0.904
7	A job where I have a lot of responsibility	0.32	0.16	0.08	0.043	0.18	0.19	0.04	0.361	0.18	0.19	0.05	0.339	0.24	0.19	0.06	0.202
8	A job that uses my skills and abilities	0.50	0.19	0.10	0.010	0.35	0.24	0.07	0.139	0.31	0.24	0.06	0.196	0.16	0.23	0.03	0.483
9	A job that people regard highly	0.04	0.14	0.01	0.755	− 0.14	0.16	− 0.04	0.363	− 0.13	0.16	− 0.04	0.410	− 0.05	0.15	− 0.01	0.734
10	A chance to learn a lot of new things	0.12	0.18	0.03	0.494	− 0.12	0.21	− 0.03	0.568	− 0.11	0.21	− 0.02	0.604	− 0.20	0.21	− 0.04	0.343

*Coef*. regression coefficient, *SE* standard error, *β* standardized regression coefficient.

^1)^Ten work values were entered into a model simultaneously; VIF = 1.25–1.62; R-squared = 0.0185004; adjusted R-squared = 0.0039164; F = 1.25 (p = 0.2548).

^2)^Adjusted for sex, race, and household income at Time 1; VIF = 1.08–1.70; R-squared = 0.0251828; adjusted R-squared = 0.0062684; F = 1.29 (p = 0.2117).

^3)^Adjusted for Model 2 + mastery at Time 1; VIF = 1.07–1.70; R-squared = 0.0735081; adjusted R-squared = 0.0541196; F = 3.70 (p < 0.0001).

**Table 10:** The association between work orientations at Time 1 with mastery at Time 2 applying linear regression analysis (N = 684).

	Bivariate associations				Multivariate analyses											
	Coef	SE	β	p	Model 1^1)^				Model 2^2)^				Model 3^3)^			
					Coef	SE	β	p	Coef	SE	β	p	Coef	SE	β	p
**Work orientations at Time 1**																
Extrinsic orientations (mean score of 4 items; #1 #2 #3 #9)	0.47	0.27	0.07	0.076	0.28	0.30	0.04	0.339	0.25	0.30	0.04	0.397	0.27	0.29	0.04	0.350
Intrinsic orientations (mean score of 6 items; #4 #5 #6 #7 #8 #10)	0.51	0.24	0.08	0.039	0.39	0.27	0.06	0.155	0.43	0.28	0.07	0.124	0.27	0.28	0.04	0.332

*Coef*. regression coefficient, *SE* standard error, *β* standardized regression coefficient.

^1)^Two orientations were entered into a model simultaneously; VIF = 1.25–1.25; R-squared = 0.0077453; adjusted R-squared = 0.0048312; F = 2.60 (p = 0.0753).

^2)^Adjusted for sex, race, and household income at Time 1; VIF = 1.06–1.31; R-squared = 0.0171631; adjusted R-squared = 0.0099151; F = 2.25 (p = 0.0476).

^3)^Adjusted for Model 2 + mastery at Time 1; VIF = 1.03–1.33; R-squared = 0.0665585; adjusted R-squared = 0.0582857; F = 7.74 (p < 0.0001).

The cross-lagged panel model analysis on the longitudinal association of work values or work orientations at Time 1 with outcomes at Time 2 (Supplemental Figs. S1, S2) generally replicated the results of the linear regression analyses (Supplemental Tables S11–S18). The work value of getting ahead at Time 1 showed a significant positive association with self-esteem at Time 2 (standardized coefficient 0.11, p = 0.014), while self-esteem at Time 1 was significantly associated with seven of the 10 work values at Time 2 (Supplemental Table S15). Self-esteem at Time 1 was significantly associated with both intrinsic and extrinsic orientations at Time 2 (Supplemental Table S16). None of the work values or work orientations at Time 1 were significantly associated with positive emotions, negative emotions, or mastery at Time 2. Positive emotions at Time 1 were significantly associated with work values of making one’s own decisions, using skills and abilities, and learning a lot of new things (Supplemental Table S11), as well as intrinsic orientations at Time 2 (Supplemental Table S12); mastery at Time 1 was significantly associated with the work value of using skills and abilities at Time 2 (Supplemental Table S17).

## Discussion

In the full model, adjusted for sociodemographic variables and self-esteem at baseline, having the work value in adolescence of getting ahead was significantly and positively associated with self-esteem in adulthood. Intrinsic orientations of work values in adolescence were significantly and positively associated with self-esteem in adulthood with adjusting for sociodemographic variables at baseline, however, its association attenuated and no longer significant after adjusting for self-esteem at baseline. Intrinsic orientations in adolescence were significantly and positively associated with positive emotions in adulthood. The work values of using one’s skills and abilities and learning new things in adolescence were significantly and positively associated with self-esteem in adulthood. The work values of employment security, having responsibilities, and using one’s skills and abilities, as well as intrinsic orientations in adolescence, were significantly and positively associated with mastery in adulthood. However, these associations were no longer significant after adjusting for the other work values or work orientations.

Having higher intrinsic orientations for work values in adolescence were significantly and positively associated with positive emotions in adulthood in a bivariate analysis, however, the significant association did not remain after adjusting for extrinsic orientations in adolescence. Intrinsic orientations in adolescence were not significantly associated with negative emotions in adulthood. It was inconsistent with previous cross-sectional studies of working adults reporting associations of current higher intrinsic values with higher work engagement^21–23^ and less job burnout^21,22^. However, such cross-sectional findings should be interpreted carefully, because positive emotions may enhance, and negative emotions may decrease, the perceptions of intrinsic values. Extrinsic orientations in adolescence were not associated with positive or negative emotions in adulthood. Previous cross-sectional studies examining associations between extrinsic work values and well-being among employees reported inconsistent findings. Some studies reported negative associations between extrinsic work orientations and well-being^15,22^, while another study reported no association between them^21^. Previous studies revealed that adolescent work orientations predict later work rewards. That is, adolescents with higher intrinsic orientations tended to choose occupations with more intrinsic work rewards (i.e. an occupation which is interesting, provides a chance to learn new skills, and provides a chance to use one’s skills and abilities), while those with higher extrinsic orientations tended to get jobs with higher hourly earnings in young adulthood^18,30^. According to the self-determination theory, competence, autonomy, and relatedness are innate, basic psychological needs for human beings, and satisfaction of these basic needs is conductive towards health and well-being^33^. Based on this theory, pursuit and attainment of intrinsic rewards were thought to facilitate the satisfaction of these basic psychological needs, while seeking and satisfying extrinsic rewards does not directly lead to their satisfaction. Furthermore, the findings of one study indicated that extrinsic work orientations inhibited the satisfaction of these basic psychological needs^15^. Therefore, according to the self-determination theory, intrinsic orientations are thought to contribute to well-being, while extrinsic orientations are not. In our study, although not statistically significant in multivariate analyses, higher intrinsic orientations in adolescence were associated with more positive emotions and less negative emotions in adulthood, while higher extrinsic orientations in adolescence were associated with more negative emotions in adulthood. The current sample size may not have had enough power to detect the impact of work orientations in adolescence on the changes in positive and negative emotions. Further research is needed to test the association between intrinsic and extrinsic orientations and mental health and well-being in a larger prospective cohort study.

In bivariate analyses, the work values in adolescence of using one’s skills and abilities (an item of intrinsic orientations) and chances to learn new things (an item of intrinsic orientations) were significantly and positively associated with self-esteem in adulthood. Having the work value in adolescence of chances of getting ahead (an item of extrinsic orientations) was significantly and positively associated with self-esteem in adulthood, even after adjusting for the baseline self-esteem score. This association was replicated by the cross-lagged panel model analysis considering work values and self-esteem at Time 1 and 2. Fetvadjiev and He^35^ reported short-term (with 1-year interval) effects of personal values on self-esteem in a longitudinal panel study. The finding is similar to those in the present study. However, our study indicated a longer-term impact of work values in adolescence on self-esteem in adulthood over 23 years. This work value of chances of getting ahead seemed to reflect the importance of one’s personal growth and correspond with the self-enhancement and openness to change values in Schwartz’s theory on personal values^1^, which were reported to be positively correlated with self-esteem in previous cross-sectional studies^6,7^. Adolescents valuing their own growth may gain higher self-esteem in young adulthood. They may strive to learn new things, develop their skills and abilities, decide what to do and how to do things by themselves, and accumulate their successful experiences with their newly acquired skills and abilities, by which they might become to perceive higher self-esteem.

Intrinsic orientations in adolescence were significantly and positively associated with self-esteem in adulthood when adjusting for the baseline sociodemographic characteristic; however, this association was no longer significant after the baseline score of self-esteem was adjusted. This is most likely because self-esteem and intrinsic orientations were already closely correlated at baseline, and it may be difficult to observe additional increases in self-esteem during follow-up. On the other hand, the work value of getting ahead in adolescence, which significantly contributed to self-esteem in adulthood even after adjusting for the baseline score of self-esteem, was a value reflecting extrinsic orientations. The finding somewhat contradicts the prediction of the self-determination theory that intrinsic orientations or motivations are associated with better self-esteem, but not extrinsic orientations^33^. Valuing good chances of getting ahead may reflect the desire for personal growth in the work domain, an intrinsic orientation, to some extent. Future research should investigate differential roles and their underlying mechanisms of intrinsic and extrinsic orientated values for positive and negative emotions and psychological resources in adulthood. It is also interesting to note that the finding is congruent with the socioanalytic theory of personality in that getting ahead is one of the most important motivations and is potentially associated with successful organizational behaviours moderated by social skill^34^. A contribution of adolescent work value of getting ahead to self-esteem could be investigated also in the light of the other theories of human motivation.

The work values in adolescence of employment security, having responsibilities, and using one’s skills and abilities, as well as intrinsic orientations, were significantly and positively associated with mastery in adulthood in bivariate analyses. Employment security seemed to be a premise of having a sense of control in one’s life. Adolescents seeking employment security may be most likely able to obtain secure job, which may have been a basis for having a sense of control in their own lives. Having the work values of taking responsibilities and using one’s skills and abilities seemed to reflect the value of openness to change in Schwartz’s theory of personal values^1^, which were also reported to be positively correlated with mastery in a previous cross-sectional study^4^. Adolescents who value responsibly using their skills and abilities in their work may become to use their skills and abilities on their own in adulthood, which may increase their mastery. Concerning the positive association between intrinsic work orientations in adolescence and mastery in adulthood, some intrinsic work rewards seem to be more attainable by workers themselves; for example, through choosing their occupation, compared to extrinsic work rewards, which sometimes require others’ evaluations. Intrinsic orientations may promote mastery through getting intrinsic rewards by themselves. However, these associations became non-significant after adjusting for the other work values or orientations. This was most likely due to the correlations between each work value or orientations in adolescence and indicated that individual work values or orientations in adolescence did not have enough effects on mastery in adulthood.

Fetvadjiev and He^35^ reported short-term bidirectional associations between personal values and self-esteem or well-being. The findings are similar, in part, to those in the present study: positive emotions, self-esteem, and mastery in adolescence were associated with some work values in adulthood. Thus, we replicated this previous finding of the effect of self-esteem or well-being on values with a longer-term perspective. Fetvadjiev and He^35^ argued that self-esteem and well-being predicted personal values better than personal values predicted self-esteem and well-being. This is consistent to some extent with our findings of cross-lagged panel analyses: we observed relatively stronger associations of positive emotions, self-esteem, and mastery in adolescence with work values in adulthood compared to those between work values in adolescence and outcomes in adulthood, while the estimated effect sizes (i.e., standardized coefficients) of the associations between the work value of getting ahead and self-esteem were almost similar for both directions. A future long-term study on values in adolescence and mental health and well-being in adulthood is needed to take into account the bidirectional effects during a life-course.

Our study has several limitations. First, we used the data of a long-term longitudinal study, and that study’s attrition rate may have caused a bias. The respondents we used in our analyses did not account for a very large proportion of the respondents in the Time 1 survey (61.9%). The respondents we used had slightly higher mastery than those who lost and attached more importance to the values of employment security, getting ahead, working with people, and making one’s own decisions at Time 1. Since mastery at Time 1 was a significant predictor of mastery at Time 2, the correlations of these work values with Time 2 mastery might be overestimated. Furthermore, the respondents analysed in the present study were more likely to be women, white, and have a high household income at Time 1 compared to those who were lost to follow-up, which limits the generalizability of our findings. Second, we did not take account of the values endorsed in the environment. There are studies indicating that particular values do not inherently contribute to well-being or self-esteem, but contribute to them depending on whether they are congruent with the values emphasized in a surrounding environment^3,7^. Furthermore, the relationships between values and self-esteem were found to be different in environments where particular values were considered more or less important^7^. Therefore, values prevalent in an environment might affect our results.

By analysing the long-term longitudinal data provided by the YDS^32^, we confirmed that work values in adolescence could predict self-esteem in adulthood. On the other hand, work values in adolescence did not predict positive emotions, negative emotions, or mastery in adulthood. Some work values reflecting the desire for personal growth in adolescence, regardless of intrinsic or extrinsic orientations, may be associated with the development of self-esteem in adulthood. The present study warrants future research on the impact of personal values in adolescence on mental health and well-being in adulthood.

## Methods

### Study design and population

The present study used data from the Youth Development Study (YDS)^32^, a long-term longitudinal study that started in 1988 and followed a random sample of students attending public schools in St. Paul, Minnesota. The baseline survey was conducted when the respondents were in the 9th grade (i.e. 14–15 years old) using a self-administered questionnaire, and follow-up surveys were conducted near-annually until 2011, when the respondents were 37–38 years old. In the present study, we used the data collected in the first survey (Time 1; 1988) and the latest survey (Time 2; 2011). We also used data from a corresponding parent survey conducted in 1988 to identify respondents’ socioeconomic statuses in adolescence.

We followed the Ethical Guidelines for Medical and Health Research Involving Human Subjects defined by Japanese Ministry of Health, Labour and Welfare and Ministry of Education, Culture, Sports, Science and Technology^36^. For the present study that used data that have already been established academically, widely utilized in research, and openly available and that do not contain information identifiable of individual persons, it should be exempted to obtain an ethical approval from an institutional review board.

### Study variables

#### Work values

Work values at Time 1 were assessed by asking the respondents to rate 10 job features from 1 (not at all important) to 4 (extremely important) based on how important they believed these features would be when they were seeking full-time employment after completing school. The job features they rated were as follows: (1) ‘good pay’, (2) ‘a steady job with little chance of being laid off’, (3) ‘good chances of getting ahead’, (4) ‘a chance to be helpful to others or useful to society’, (5) ‘a chance to work with people rather than things’, (6) ‘a chance to make my own decisions’, (7) ‘a job where I have a lot of responsibility’, (8) ‘a job that uses my skills and abilities’, (9) ‘a job that people regard highly’, and (10) ‘a chance to learn a lot of new things’. We averaged the ratings of Items 1–3 and 9 to create a scale of extrinsic orientations and the ratings of Items 4–8 and 10 to create a scale of intrinsic orientations, based on previous studies using the same data^17,20^. This two-dimensional conceptualization of work values was also supported in our study subjects. We conducted an exploratory factor analysis with principal-components factor estimates and promax rotation using the responses at Time 1. As a result, the first two factors had eigenvalues higher than 1 (3.44 and 1.47, respectively). The first factor was loaded by six items corresponding to intrinsic orientations (explaining 30.2% of the variance), and the second factor was loaded by four items corresponding to extrinsic orientations (explaining 26.8% of the variance). The Cronbach’s alpha coefficients of the four extrinsic orientations items and the six intrinsic orientations items were 0.62 and 0.77, respectively.

Work values at Time 2 were assessed with the same scale by asking the respondents how important these things were to them when they were looking for work. Cronbach’s alpha coefficients of the four extrinsic orientations items and the six intrinsic orientations items were 0.66 and 0.80, respectively.

#### Mental health and well-being

Mental health and well-being during the previous month were assessed with nine items derived from the Health Insurance Study (HIS) mental health battery^37^, which included: (1) ‘have you felt that the future looks hopeful and promising’, (2) ‘have you been under any strain, stress, or pressure’, (3) ‘have you generally enjoyed the things you do’, (4) ‘have you felt calm and peaceful’, (5) ‘have you felt downhearted and blue’, (6) ‘have you been moody or brooded about things’, (7) ‘have you felt cheerful or light-hearted’, (8) ‘have you felt depressed’, and (9) ‘have you been in low or very low spirits’. The items were rated on a 5-point Likert scale ranging from 1 (none of the time) to 5 (all of the time).

To examine the differential impacts of work values on negative and positive emotions separately, we developed two separate scales, one to measure each type of emotions. After Items 2, 5, 6, 8, and 9 were reverse coded, we conducted an exploratory factor analysis with principal-components factor estimates and promax rotation using the responses at Time 2. As a result, the first two factors had eigenvalues higher than 1 (4.74 and 1.24, respectively). The first factor was loaded by Items 2, 5, 6, 8, and 9 (explaining 47.3% of the variance), and the second factor was loaded by Items 1, 3, 4, and 7 (explaining 39.7% of the variance). We developed two scales corresponding to this two-factor structure and named them the ‘negative emotion scale’ and ‘positive emotion scale’. As for the negative emotion scale, item scores were coded back to reflect higher scores indicating higher negative emotions and summed up, the total scale score of which ranged from 5 to 25. Cronbach’s alpha coefficients of the five items at Time 1 and Time 2 were 0.84 and 0.89, respectively. As for the positive emotion scale, the total scale score ranged from 4 to 20, with a higher score indicating higher positive emotions. Cronbach’s alpha coefficients of the four items at Time 1 and Time 2 were 0.68 and 0.79, respectively.

#### Self-esteem

Self-esteem was assessed using seven of the 10 items of the Rosenberg Self-esteem Scale^38^. The items are rated on a 4-point Likert scale ranging from 1 (strongly disagree) to 4 (strongly agree). Four of the seven items were reverse coded and summed, with a total score ranging from 7 to 28. Higher scores indicated higher self-esteem. The items used in this study included: (1) ‘I feel I have a number of good qualities’, (2) ‘I certainly feel useless at times’, (3) ‘I feel I do not have much to be proud of’, (4) ‘I take a positive attitude toward myself’, (5) ‘on the whole, I am satisfied with myself’, (6) ‘at times I think I am no good at all’, and (7) ‘I wish I could have more respect for myself’. Cronbach’s alpha coefficients of the seven items at Time 1 and Time 2 were 0.80 and 0.85, respectively.

#### Mastery

Mastery was assessed using the 7-item Pearlin Mastery Scale^39^. Items are rated on a 4-point Likert scale ranging from 1 (strongly disagree) to 4 (strongly agree). Five of the 7 items were reverse coded and summed, with total scores ranging from 7 to 28. Higher scores indicated higher mastery. The items included: (1) ‘There is really no way I can solve some of the problems I have’, (2) ‘Sometimes I feel that I’m being pushed around in life’, (3) ‘I have little control over things that happen to me’, (4) ‘I can do just about anything I really set my mind to’, (5) ‘What happens to me in the future mostly depends on me’, (6) ‘I often feel helpless in dealing with problems of life’, and (7) ‘There is little I can do to change many of the important things in my life’. Cronbach’s alpha coefficients of the scale at Time 1 and Time 2 were 0.67 and 0.83, respectively.

#### Sociodemographic characteristics

The sociodemographic characteristics included in this study were sex, race, and household income at Time 1, and educational attainment, employment status, marital status, and household income at Time 2. The information on race was dichotomized as white and non-white (Black, Hispanic, Southeast Asian, other Asian, Native American, mixed race, or other). Information on total annual household income at Time 1 was obtained using the parents’ survey with 13 categories (1. under $5,000; 2. $5,000–9,999; 3. $10,000–14,999; 4. $15,000–19,999; 5. $20,000–29,999; 6. $30,000–39,999; …; 12. $90,000–99,999; 13. $100,000 or more in USD) and used as a continuous variable (1–13).

### Statistical analysis

We calculated descriptive statistics for sociodemographic characteristics, work values, and mental health and well-being and psychological resources at Time 1 and Time 2 for the respondents used in the present study. We then compared sociodemographic characteristics, work values, mental health and well-being and psychological resources at Time 1 of respondents used in the present study with those of the individuals who responded to the Time 1 survey, but were not included in the present study, due to non-response to the Time 2 survey or missing information.

We examined the relationships between work values at Time 1 and four outcomes (i.e. positive emotions, negative emotions, self-esteem, and mastery at Time 2) separately, using linear regression analyses. First, we examined bivariate associations of each work value with each outcome. Second, we examined correlations between work values and each outcome by including all 10 work values simultaneously in a model (Model 1). Third, we examined correlations between work values and each outcome, adjusting for sex, race, and household income at Time 1 (Model 2). Fourth, we added positive emotions, negative emotions, self-esteem, or mastery at Time 1 to the respective models to examine whether the correlations of work values with each outcome remained significant (Model 3). We also examined the relationships of extrinsic and intrinsic orientations at Time 1 with each outcome at Time 2 by the same procedure.

We used the responses of those who participated both in the Time 1 and Time 2 survey. To handle missing information, we used multiple imputation with all the study variables mentioned above. We created 20 datasets using multivariate normal regression.

We replicated longitudinal associations of work values (10 work values or extrinsic and intrinsic orientations) at Time 1 with each outcome (positive emotions, negative emotions, self-esteem, or mastery) at Time 2 by applying a cross-lagged panel model with incorporating the outcome at Time 1 and work values at Time 2, using Structural Equation Modelling, where a saturated-model was assumed, including every direct association among the variables in the model (Supplemental Figs. S1, S2). We used Full Information Maximum Likelihood to handle missing information.

All statistical analyses were performed using Stata 15 for Windows (StataCorp LP, College Station, TX, USA). Statistical significance was set at 0.05 and all tests were two-tailed.

## Supplementary information

Supplementary file1
