# Supplementary material for: Longitudinal association between adolescent work values and mental health and well-being in adulthood: a 23-year prospective cohort study
Source: Sci Rep. 2020 Aug 11;10:13547. doi: 10.1038/s41598-020-70507-y (PMC7419292; doi:10.1038/s41598-020-70507-y)
Supplement: Supplementary file 1 — Supplementary file1 [file 41598_2020_70507_MOESM1_ESM.pdf]

supplement file

Longitudinal association between adolescent work values and mental health and well-being in adulthood: A 23-year prospective cohort study

Maiko Fukasawa[1], Kazuhiro Watanabe[1], Daisuke Nishi[1], Norito Kawakami[1]*

[1]Department of Mental Health, Graduate School of Medicine, The University of Tokyo

*Corresponding author
Norito Kawakami
Department of Mental Health, Graduate School of Medicine, the University of Tokyo
7-3-1 Hongo Bunkyo-ku, Tokyo 113-0033 Japan
Tel: +81-3-5841-3521, Fax: +81-3-5841-3392
E-mail: nkawakami@m.u-tokyo.ac.jp

Supplemental Table 1. Socio-demographic characteristics, work values, and mental health and well-being and psychological resources at Time 1 of the study subjects and those who lost to follow-up (n = 1105).

		Subjects in this study (n = 684)[a]		Individuals who lost to follow-up (n = 421)[b]		
		n / mean	% / SD	n / mean	% / SD	p
Sex						
	Men	278	41.3	242	58.7	<0.001
	Women	395	58.7	170	41.3	
Race						
	White	498	74.3	223	53.9	<0.001
	Others	172	25.7	191	46.1	
Household income at Time 1						
	Under $20,000	179	27.5	160	43.0	<0.001
	$20,000-40,000	221	34.0	120	32.3	
	$40,000 or more	251	38.6	92	24.7	
Work values at Time 1						
1	Good pay	3.67	0.53	3.65	0.58	0.609
2	A steady job, with little chance of being laid off	3.59	0.70	3.49	0.81	0.018
3	Good chances of getting ahead	3.63	0.60	3.52	0.72	0.008
4	A chance to be helpful to others or useful to society	3.02	0.82	3.02	0.84	0.921
5	A chance to work with people rather than things	3.00	0.86	2.89	0.92	0.041
6	A chance to make my own decisions	3.19	0.73	3.08	0.83	0.022
7	A job where I have a lot of responsibility	2.97	0.80	2.98	0.87	0.878
8	A job that uses my skills and abilities	3.53	0.66	3.47	0.72	0.180
9	A job that people regard highly	3.04	0.90	3.09	0.89	0.349
10	A chance to learn a lot of new things	3.38	0.69	3.37	0.74	0.924
Extrinsic orientations (Mean score of 4 items; #1 #2 #3 #9)		3.48	0.47	3.44	0.51	0.171
Intrinsic orientations (Mean score of 6 items; #4 #5 #6 #7 #8 #10)		3.19	0.52	3.14	0.56	0.178
Mental health and well-being and psychological resources at Time 1						
	Positive emotions	13.53	2.47	13.50	2.75	0.836
	Negative emotions	12.91	3.82	13.08	3.94	0.492
	Self-esteem	20.02	3.40	19.76	3.48	0.241
	Mastery	20.18	3.03	19.66	3.18	0.008

SD: standard deviation

[a] Since subjects in this study had several missing values, the sums of each cell are not equal to 684.

[b] It includes 4 individuals who were removed from the study subjects because of non-response to any item on work values at Time 1. Since the respondents had several missing values, the sums of each cell are not equal to 421.

Supplemental Table 2. Correlations between each work value at Time 1

		Pearson's correlation coefficient										
		2	3	4	5	6	7	8	9	10	Extrinsic	Intrinsic
1	Good pay	0.375	0.315	0.069	0.116	0.209	0.170	0.225	0.254	0.158	0.638	0.216
2	A steady job, with little chance of being laid off		0.440	0.090	0.035	0.290	0.215	0.280	0.202	0.103	0.707	0.233
3	Good chances of getting ahead			0.229	0.178	0.303	0.215	0.344	0.300	0.252	0.707	0.361
4	A chance to be helpful to others or useful to society				0.568	0.232	0.358	0.306	0.193	0.342	0.219	0.707
5	A chance to work with people rather than things					0.306	0.321	0.298	0.244	0.324	0.219	0.715
6	A chance to make my own decisions						0.431	0.403	0.329	0.310	0.423	0.643
7	A job where I have a lot of responsibility							0.428	0.293	0.355	0.333	0.710
8	A job that uses my skills and abilities								0.245	0.399	0.392	0.663
9	A job that people regard highly									0.246	0.716	0.376
10	A chance to learn a lot of new things										0.282	0.647
	Extrinsic orientations (Mean score of 4 items; #1 #2 #3 #9)											0.441
	Intrinsic orientations (Mean score of 6 items; #4 #5 #6 #7 #8 #10)											

Supplemental Table 3. The association between work values at Time 1 with positive emotions at Time 2 applying linear regression analysis among the respondents without missing information (N = 622)

		Bivariate associations				Multivariate analyses											
						Model 1 1)				Model 2 2)				Model 3 3)			
		Coef.	SE	β	p	Coef.	SE	β	p	Coef.	SE	β	p	Coef.	SE	β	p
Work values at Time 1																	
1	Good pay	-0.08	0.19	-0.02	0.666	-0.20	0.21	-0.04	0.346	-0.20	0.21	-0.04	0.352	-0.22	0.21	-0.05	0.284
2	A steady job, with little chance of being laid off	-0.09	0.15	-0.02	0.542	-0.25	0.18	-0.07	0.164	-0.27	0.18	-0.07	0.136	-0.24	0.18	-0.06	0.183
3	Good chances of getting ahead	0.28	0.17	0.07	0.098	0.27	0.20	0.06	0.175	0.29	0.20	0.07	0.158	0.23	0.20	0.06	0.242
4	A chance to be helpful to others or useful to society	0.19	0.12	0.06	0.126	0.04	0.16	0.01	0.791	0.08	0.16	0.03	0.603	0.13	0.16	0.04	0.429
5	A chance to work with people rather than things	0.18	0.12	0.06	0.126	0.04	0.15	0.01	0.806	0.04	0.15	0.01	0.794	-0.02	0.15	-0.01	0.882
6	A chance to make my own decisions	0.14	0.14	0.04	0.313	-0.02	0.17	-0.01	0.892	-0.03	0.17	-0.01	0.841	-0.08	0.17	-0.02	0.646
7	A job where I have a lot of responsibility	0.17	0.13	0.05	0.171	0.04	0.15	0.01	0.789	0.05	0.15	0.01	0.767	0.00	0.15	0.00	1.000
8	A job that uses my skills and abilities	0.29	0.15	0.08	0.056	0.19	0.19	0.05	0.309	0.18	0.19	0.05	0.344	0.10	0.19	0.03	0.590
9	A job that people regard highly	0.20	0.11	0.07	0.076	0.15	0.12	0.05	0.237	0.15	0.12	0.06	0.221	0.19	0.12	0.07	0.134
10	A chance to learn a lot of new things	0.24	0.14	0.07	0.097	0.08	0.17	0.02	0.625	0.08	0.17	0.02	0.609	0.06	0.16	0.02	0.708

Coef.: regression coefficient; SE: standard error; β: standardized regression coefficient

1) Ten work values were entered into a model simultaneously; VIF = 1.25-1.63; R-squared = 0.0178; adjusted R-squared = 0.0018; F = 1.11 (p = 0.3524)

2) Adjusted for sex, race, and household income at Time 1; VIF = 1.09-1.70; R-squared = 0.4499; adjusted R-squared = -0.0000; F = 1.00 (p = 0.4499)

3) Adjusted for Model 2 + positive emotions at Time 1 (N = 613); VIF = 1.05-1.71; R-squared = 0.0492; adjusted R-squared = 0.0492; F = 2.21 (p = 0.0066)

Supplemental Table 4. The association between work orientations at Time 1 with positive emotions at Time 2 applying linear regression analysis among the respondents without missing information (N = 622)

	Bivariate associations				Multivariate analyses											
					Model 1 [1)]				Model 2 [2)]				Model 3 [3)]			
	Coef.	SE	β	p	Coef.	SE	β	p	Coef.	SE	β	p	Coef.	SE	β	p
Work orientations at Time 1																
Extrinsic orientations (Mean score of 4 items; #1 #2 #3 #9)	0.22	0.21	0.04	0.302	0.02	0.24	0.00	0.917	0.01	0.24	0.00	0.963	0.00	0.23	0.00	0.983
Intrinsic orientations (Mean score of 6 items; #4 #5 #6 #7 #8 #10)	0.43	0.19	0.09	0.028	0.42	0.21	0.09	0.053	0.45	0.22	0.09	0.039	0.25	0.22	0.05	0.249

Coef.: regression coefficient; SE: standard error

1) Two orientations were entered into a model simultaneously; VIF = 1.22-1.22; R-squared = 0.0078; adjusted R-squared = 0.0046; F = 2.42 (p = 0.0896)

2) Adjusted for sex, race, and household income at Time 1; VIF = 1.05-1.29; R-squared = 0.0100; adjusted R-squared = 0.0019; F = 1.24 (p = 0.2882)

3) Adjusted for Model 2 + positive emotions at Time 1 (N = 613); VIF = 1.05-1.33; R-squared = 0.0377; adjusted R-squared = 0.0281; F = 3.95 (p = 0.0007)

Supplemental Table 5. The association between work values at Time 1 with negative emotions at Time 2 applying linear regression analysis among the respondents without missing information (N = 622)

		Bivariate associations				Multivariate analyses											
						Model 1 1)				Model 2 2)				Model 3 3)			
		Coef.	SE	β	p	Coef.	SE	β	p	Coef.	SE	β	p	Coef.	SE	β	p
Work values at Time 1																	
1	Good pay	0.37	0.28	0.05	0.179	0.37	0.31	0.05	0.232	0.39	0.31	0.06	0.210	0.37	0.31	0.05	0.241
2	A steady job, with little chance of being laid off	0.27	0.22	0.05	0.227	0.31	0.26	0.06	0.246	0.33	0.27	0.06	0.217	0.36	0.27	0.06	0.182
3	Good chances of getting ahead	-0.14	0.25	-0.02	0.584	-0.36	0.30	-0.06	0.230	-0.39	0.30	-0.06	0.190	-0.43	0.30	-0.07	0.158
4	A chance to be helpful to others or useful to society	-0.02	0.18	-0.01	0.891	0.13	0.23	0.03	0.579	0.06	0.24	0.01	0.797	0.03	0.24	0.01	0.898
5	A chance to work with people rather than things	-0.11	0.17	-0.03	0.509	-0.13	0.22	-0.03	0.570	-0.15	0.22	-0.03	0.503	-0.12	0.22	-0.03	0.585
6	A chance to make my own decisions	-0.04	0.21	-0.01	0.840	-0.06	0.25	-0.01	0.799	-0.05	0.25	-0.01	0.833	0.02	0.26	0.00	0.928
7	A job where I have a lot of responsibility	-0.01	0.19	0.00	0.971	0.03	0.23	0.01	0.904	0.02	0.23	0.00	0.937	-0.02	0.23	0.00	0.927
8	A job that uses my skills and abilities	-0.14	0.23	-0.02	0.549	-0.14	0.28	-0.02	0.625	-0.12	0.28	-0.02	0.670	-0.09	0.28	-0.02	0.742
9	A job that people regard highly	0.13	0.16	0.03	0.421	0.17	0.18	0.04	0.347	0.17	0.19	0.04	0.357	0.10	0.19	0.02	0.583
10	A chance to learn a lot of new things	-0.20	0.21	-0.04	0.352	-0.19	0.25	-0.04	0.430	-0.20	0.25	-0.04	0.421	-0.17	0.25	-0.03	0.497

Coef.: regression coefficient; SE: standard error; β: standardized regression coefficient

1) Ten work values were entered into a model simultaneously; VIF = 1.25-1.63; R-squared = 0.0109; adjusted R-squared = -0.0053; F = 0.67 (p = 0.7498)

2) Adjusted for sex, race, and household income at Time 1; VIF = 1.09-1.70; R-squared = 0.0161; adjusted R-squared = -0.0049; F = 0.77 (p = 0.6971)

3) Adjusted for Model 2 + negative emotions at Time 1 (N = 605); VIF = 1.04-1.74; R-squared = 0.0379; adjusted R-squared = 0.0150; F = 1.66 (p = 0.0601)

Supplemental Table 6. The association between work orientations at Time 1 with negative emotions at Time 2 applying linear regression analysis among the respondents without missing information (N = 622)

	Bivariate associations				Multivariate analyses											
					Model 1 [1)]				Model 2 [2)]				Model 3 [3)]			
	Coef.	SE	β	p	Coef.	SE	β	p	Coef.	SE	β	p	Coef.	SE	β	p
Work orientations at Time 1																
Extrinsic orientations (Mean score of 4 items; #1 #2 #3 #9)	0.32	0.32	0.04	0.305	0.50	0.35	0.06	0.155	0.53	0.35	0.07	0.133	0.44	0.35	0.06	0.215
Intrinsic orientations (Mean score of 6 items; #4 #5 #6 #7 #8 #10)	-0.18	0.29	-0.02	0.541	-0.37	0.32	-0.05	0.245	-0.48	0.33	-0.07	0.145	-0.41	0.33	-0.06	0.206

Coef.: regression coefficient; SE: standard error

1) Two orientations were entered into a model simultaneously; VIF = 1.22-1.22; R-squared = 0.0039; adjusted R-squared = 0.0007; F = 1.20 (p = 0.3009)

2) Adjusted for sex, race, and household income at Time 1; VIF = 1.05-1.29; R-squared = 0.0088; adjusted R-squared = 0.0007; F = 1.09 (p = 0.3644)

3) Adjusted for Model 2 + negative emotions at Time 1 (N = 605); VIF = 1.03-1.28; R-squared = 0.0305; adjusted R-squared = 0.0207; F = 3.13 (p = 0.0050)

Supplemental Table 7. The association between work values at Time 1 with self-esteem at Time 2 applying linear regression analysis among the respondents without missing information (N = 624)

		Bivariate associations				Multivariate analyses											
						Model 1 [1)]				Model 2 [2)]				Model 3 [3)]			
		Coef.	SE	β	p	Coef.	SE	β	p	Coef.	SE	β	p	Coef.	SE	β	p
Work values at Time 1																	
1	Good pay	0.25	0.27	0.04	0.362	0.00	0.30	0.00	0.991	-0.02	0.30	0.00	0.950	-0.01	0.30	0.00	0.986
2	A steady job, with little chance of being laid off	0.12	0.22	0.02	0.573	-0.25	0.26	-0.05	0.331	-0.28	0.26	-0.05	0.276	-0.24	0.25	-0.04	0.352
3	Good chances of getting ahead	0.71	0.24	0.12	0.004	0.72	0.29	0.12	0.014	0.76	0.29	0.12	0.009	0.85	0.29	0.14	0.003
4	A chance to be helpful to others or useful to society	-0.01	0.18	0.00	0.969	-0.44	0.22	-0.10	0.052	-0.36	0.23	-0.08	0.119	-0.38	0.22	-0.09	0.094
5	A chance to work with people rather than things	0.27	0.17	0.06	0.113	0.26	0.21	0.06	0.223	0.28	0.21	0.07	0.187	0.13	0.21	0.03	0.541
6	A chance to make my own decisions	0.38	0.20	0.08	0.060	0.12	0.24	0.02	0.629	0.10	0.24	0.02	0.674	0.18	0.24	0.04	0.451
7	A job where I have a lot of responsibility	0.31	0.18	0.07	0.092	0.14	0.22	0.03	0.526	0.15	0.22	0.03	0.492	0.08	0.22	0.02	0.728
8	A job that uses my skills and abilities	0.53	0.22	0.10	0.017	0.27	0.27	0.05	0.318	0.24	0.27	0.04	0.374	0.18	0.27	0.03	0.498
9	A job that people regard highly	0.10	0.16	0.02	0.542	-0.15	0.18	-0.04	0.407	-0.14	0.18	-0.04	0.421	-0.10	0.18	-0.02	0.584
10	A chance to learn a lot of new things	0.45	0.21	0.09	0.030	0.25	0.24	0.05	0.291	0.26	0.24	0.05	0.279	0.19	0.23	0.04	0.414

Coef.: regression coefficient; SE: standard error

1) Ten work values were entered into a model simultaneously; VIF = 1.24-1.63; R-squared = 0.0285; adjusted R-squared = 0.0126; F = 1.80 (p = 0.0578)

2) Adjusted for sex, race, and household income at Time 1; VIF = 1.09-1.70; R-squared = 0.0361; adjusted R-squared = 0.0155; F = 1.76 (p = 0.0466)

3) Adjusted for Model 2 + self-esteem at Time 1 (N = 598); VIF = 1.08-1.71; R-squared = 0.1095; adjusted R-squared = 0.0881; F = 5.12 (p < 0.0001)

Supplemental Table 8. The association between work orientations at Time 1 with self-esteem at Time 2 applying linear regression analysis among the respondents without missing information (N = 624)

	Bivariate associations				Multivariate analyses											
					Model 1 [1)]				Model 2 [2)]				Model 3 [3)]			
	Coef.	SE	β	p	Coef.	SE	β	p	Coef.	SE	β	p	Coef.	SE	β	p
Work orientations at Time 1																
Extrinsic orientations (Mean score of 4 items; #1 #2 #3 #9)	0.52	0.31	0.07	0.094	0.26	0.34	0.03	0.441	0.23	0.34	0.03	0.508	0.45	0.34	0.06	0.186
Intrinsic orientations (Mean score of 6 items; #4 #5 #6 #7 #8 #10)	0.64	0.28	0.09	0.022	0.54	0.31	0.08	0.081	0.67	0.32	0.10	0.034	0.34	0.31	0.05	0.280

Coef.: regression coefficient; SE: standard error; β: standardized regression coefficient

1) Two orientations were entered into a model simultaneously; VIF = 1.22-1.22; R-squared = 0.0094; adjusted R-squared = -0.0062; F = 2.93 (p = 0.0539)

2) Adjusted for sex, race, and household income at Time 1; VIF = 1.05-1.29; R-squared = 0.0183; adjusted R-squared = 0.0104; F = 2.31 (p = 0.0430)

3) Adjusted for Model 2 + self-esteem at Time 1 (N = 598); VIF = 1.07-1.30; R-squared = 0.0913; adjusted R-squared = 0.0820; F = 9.89 (p < 0.0001)

Supplemental Table 9. The association between work values at Time 1 with mastery at Time 2 applying linear regression analysis among the respondents without missing information (N = 618)

		Bivariate associations				Multivariate analyses											
						Model 1 [1)]				Model 2 [2)]				Model 3 [3)]			
		Coef.	SE	β	p	Coef.	SE	β	p	Coef.	SE	β	p	Coef.	SE	β	p
Work values at Time 1																	
1	Good pay	0.23	0.25	0.04	0.355	-0.05	0.27	-0.01	0.866	-0.03	0.28	0.00	0.915	-0.05	0.28	-0.01	0.870
2	A steady job, with little chance of being laid off	0.46	0.20	0.09	0.019	0.33	0.23	0.07	0.155	0.29	0.24	0.06	0.222	0.29	0.23	0.06	0.208
3	Good chances of getting ahead	0.37	0.22	0.07	0.093	0.14	0.26	0.03	0.592	0.17	0.27	0.03	0.521	0.06	0.26	0.01	0.809
4	A chance to be helpful to others or useful to society	0.05	0.16	0.01	0.748	-0.19	0.21	-0.05	0.345	-0.14	0.21	-0.03	0.511	-0.14	0.21	-0.03	0.511
5	A chance to work with people rather than things	0.19	0.15	0.05	0.210	0.20	0.20	0.05	0.313	0.19	0.20	0.05	0.322	0.18	0.19	0.05	0.347
6	A chance to make my own decisions	0.34	0.18	0.07	0.066	0.11	0.22	0.02	0.635	0.08	0.22	0.02	0.704	0.05	0.22	0.01	0.808
7	A job where I have a lot of responsibility	0.31	0.17	0.07	0.065	0.18	0.20	0.04	0.379	0.18	0.20	0.04	0.368	0.24	0.20	0.06	0.232
8	A job that uses my skills and abilities	0.44	0.20	0.09	0.032	0.26	0.25	0.05	0.293	0.22	0.25	0.04	0.364	0.11	0.25	0.02	0.665
9	A job that people regard highly	0.06	0.15	0.02	0.666	-0.12	0.16	-0.03	0.465	-0.11	0.16	-0.03	0.487	-0.03	0.16	-0.01	0.876
10	A chance to learn a lot of new things	0.10	0.19	0.02	0.613	-0.13	0.22	-0.03	0.564	-0.12	0.22	-0.02	0.595	-0.21	0.22	-0.05	0.324

Coef.: regression coefficient; SE: standard error; β: standardized regression coefficient

1) Ten work values were entered into a model simultaneously; VIF = 1.24-1.63; R-squared = 0.0181; adjusted R-squared = 0.0019; F = 1.12 (p = 0.3444)

2) Adjusted for sex, race, and household income at Time 1; VIF = 1.09-1.70; R-squared = 0.0247; adjusted R-squared = 0.0037; F = 1.18 (p = 0.2914)

3) Adjusted for Model 2 + mastery at Time 1 (N = 605); VIF = 1.06-1.71; R-squared = 0.0680; adjusted R-squared = 0.0459; F = 3.07 (p = 0.0001)

Supplemental Table 10. The association between work orientations at Time 1 with mastery at Time 2 applying linear regression analysis among the respondents without missing information (N = 618)

	Bivariate associations				Multivariate analyses											
					Model 1 [1)]				Model 2 [2)]				Model 3 [3)]			
	Coef.	SE	β	p	Coef.	SE	β	p	Coef.	SE	β	p	Coef.	SE	β	p
Work orientations at Time 1																
Extrinsic orientations (Mean score of 4 items; #1 #2 #3 #9)	0.52	0.28	0.07	0.066	0.35	0.31	0.05	0.261	0.32	0.31	0.05	0.304	0.32	0.31	0.05	0.291
Intrinsic orientations (Mean score of 6 items; #4 #5 #6 #7 #8 #10)	0.49	0.26	0.08	0.056	0.35	0.28	0.06	0.217	0.40	0.29	0.06	0.172	0.25	0.29	0.04	0.385

Coef.: regression coefficient; SE: standard error

1) Two orientations were entered into a model simultaneously; VIF = 1.23-1.23; R-squared = 0.0079; adjusted R-squared = 0.0047; F = 2.46 (p = 0.0861)

2) Adjusted for sex, race, and household income at Time 1; VIF = 1.06-1.30; R-squared = 0.0171; adjusted R-squared = 0.0091; F = 2.13 (p = 0.0602)

3) Adjusted for Model 2 + mastery at Time 1 (N = 605); VIF = 1.03-1.30; R-squared = 0.0608; adjusted R-squared = 0.0513; F = 6.45 (p < 0.0001)

Supplemental Table 11. Associations between work values and positive emotions at Time 1 with work values and positive emotions at Time 2 in the cross-lagged panel model (N = 684)

		**Work values at Time 2**																	
		Work value 1			Work value 2			Work value 3			Work value 4			Work value 5			Work value 6		
		Coef.	SE	p	Coef.	SE	p	Coef.	SE	p	Coef.	SE	p	Coef.	SE	p	Coef.	SE	p
Work values at Time 1																			
1	Good pay	0.17	0.04	<0.001	0.14	0.04	0.001	0.10	0.04	0.015	0.04	0.04	0.350	-0.05	0.04	0.244	-0.01	0.04	0.792
2	A steady job, with little chance of being laid off	0.05	0.04	0.290	0.04	0.05	0.376	-0.03	0.04	0.556	0.01	0.05	0.836	0.03	0.04	0.458	0.02	0.05	0.643
3	Good chances of getting ahead	0.00	0.04	0.962	-0.12	0.05	0.008	-0.02	0.04	0.698	-0.06	0.05	0.186	-0.06	0.04	0.214	0.03	0.05	0.580
4	A chance to be helpful to others or useful to society	0.04	0.05	0.451	0.03	0.05	0.570	0.01	0.05	0.764	0.17	0.05	0.001	0.11	0.05	0.026	0.07	0.05	0.188
5	A chance to work with people rather than things	0.00	0.05	0.952	-0.02	0.05	0.659	0.00	0.05	0.959	0.01	0.05	0.800	0.16	0.05	0.001	-0.03	0.05	0.604
6	A chance to make my own decisions	-0.04	0.04	0.378	-0.07	0.05	0.144	-0.03	0.05	0.506	0.04	0.05	0.335	0.01	0.04	0.816	0.05	0.05	0.312
7	A job where I have a lot of responsibility	0.01	0.05	0.855	0.04	0.05	0.342	0.09	0.05	0.052	-0.07	0.05	0.120	-0.04	0.04	0.339	-0.03	0.05	0.494
8	A job that uses my skills and abilities	-0.02	0.05	0.666	-0.04	0.05	0.447	-0.07	0.05	0.144	0.03	0.05	0.519	0.03	0.05	0.511	0.00	0.05	0.948
9	A job that people regard highly	0.02	0.04	0.648	0.00	0.04	0.913	-0.03	0.04	0.443	-0.01	0.04	0.785	0.03	0.04	0.473	0.09	0.04	0.028
10	A chance to learn a lot of new things	0.00	0.04	0.919	0.07	0.04	0.128	0.09	0.04	0.030	0.03	0.04	0.507	-0.03	0.04	0.551	-0.03	0.04	0.567
Positive emotions at Time 1		0.05	0.04	0.159	0.01	0.04	0.765	0.07	0.04	0.066	0.01	0.04	0.731	0.05	0.04	0.171	0.10	0.04	0.013

		**Work values at Time 2**												**Positive emotions at Time 2**		
		Work value 7			Work value 8			Work value 9			Work value 10					
		Coef.	SE	p	Coef.	SE	p	Coef.	SE	p	Coef.	SE	p	Coef.	SE	p
Work values at Time 1																
1	Good pay	0.01	0.04	0.870	-0.01	0.04	0.765	0.05	0.04	0.234	-0.02	0.04	0.598	-0.03	0.04	0.418
2	A steady job, with little chance of being laid off	-0.01	0.05	0.786	0.04	0.04	0.400	-0.11	0.04	0.014	-0.04	0.05	0.369	-0.06	0.05	0.193
3	Good chances of getting ahead	-0.06	0.05	0.175	0.01	0.04	0.900	-0.06	0.04	0.174	-0.06	0.05	0.191	0.06	0.05	0.156

4	A chance to be helpful to others or useful to society	0.04	0.05	0.426	-0.03	0.05	0.607	0.07	0.05	0.123	0.08	0.05	0.108	0.02	0.05	0.640
5	A chance to work with people rather than things	-0.02	0.05	0.673	-0.06	0.05	0.251	-0.09	0.05	0.071	-0.06	0.05	0.220	0.00	0.05	0.973
6	A chance to make my own decisions	-0.04	0.05	0.448	0.02	0.05	0.714	0.01	0.04	0.759	0.03	0.05	0.578	-0.03	0.05	0.572
7	A job where I have a lot of responsibility	0.09	0.05	0.064	-0.04	0.05	0.350	-0.04	0.05	0.384	-0.01	0.05	0.759	0.02	0.05	0.619
8	A job that uses my skills and abilities	0.00	0.05	0.998	0.11	0.05	0.017	-0.01	0.05	0.871	0.05	0.05	0.302	0.04	0.05	0.443
9	A job that people regard highly	0.02	0.04	0.650	0.06	0.04	0.185	0.22	0.04	<0.001	0.06	0.04	0.180	0.05	0.04	0.274
10	A chance to learn a lot of new things	0.01	0.04	0.803	-0.04	0.04	0.324	0.04	0.04	0.314	0.03	0.04	0.523	-0.01	0.04	0.842
Positive emotions at Time 1		0.07	0.04	0.091	0.10	0.04	0.007	0.04	0.04	0.260	0.09	0.04	0.017	0.18	0.04	<0.001

Coef.: coefficient; SE: standard error

All coefficients were standardized.

Supplemental Table 12. Associations between work orientations and positive emotions at Time 1 with work orientations and positive emotions at Time 2 in the cross-lagged panel model (N = 684)

	Work orientations at Time 2						Positive emotions at Time 2		
	Extrinsic orientations			Intrinsic orientations					
	Coef.	SE	p	Coef.	SE	p	Coef.	SE	p
Work orientations at Time 1									
Extrinsic orientations	0.08	0.04	0.045	-0.01	0.04	0.893	0.00	0.04	0.988
Intrinsic orientations	0.03	0.04	0.559	0.11	0.04	0.012	0.05	0.04	0.287
Positive emotions at Time 1	0.06	0.04	0.095	0.10	0.04	0.013	0.19	0.04	<0.001

Coef.: coefficient; SE: standard error

All coefficients were standardized.

Supplemental Table 13. Associations between work values and negative emotions at Time 1 with work values and negative emotions at Time 2 in the cross-lagged panel model (N = 684)

		**Work values at Time 2**																	
		Work value 1			Work value 2			Work value 3			Work value 4			Work value 5			Work value 6		
		Coef.	SE	p	Coef.	SE	p	Coef.	SE	p	Coef.	SE	p	Coef.	SE	p	Coef.	SE	p
Work values at Time 1																			
1	Good pay	0.17	0.04	<0.001	0.14	0.04	0.001	0.10	0.04	0.015	0.04	0.04	0.358	-0.05	0.04	0.243	-0.01	0.04	0.783
2	A steady job, with little chance of being laid off	0.05	0.04	0.290	0.04	0.05	0.362	-0.03	0.04	0.505	0.01	0.05	0.857	0.03	0.04	0.505	0.02	0.05	0.708
3	Good chances of getting ahead	0.00	0.04	0.962	-0.12	0.05	0.008	-0.02	0.04	0.720	-0.06	0.05	0.195	-0.05	0.04	0.226	0.03	0.05	0.561
4	A chance to be helpful to others or useful to society	0.04	0.05	0.453	0.03	0.05	0.587	0.02	0.05	0.713	0.17	0.05	0.001	0.11	0.05	0.024	0.07	0.05	0.166
5	A chance to work with people rather than things	0.00	0.05	0.986	-0.02	0.05	0.670	0.01	0.05	0.884	0.01	0.05	0.800	0.16	0.05	0.001	-0.02	0.05	0.694
6	A chance to make my own decisions	-0.04	0.04	0.417	-0.06	0.05	0.154	-0.03	0.05	0.533	0.04	0.05	0.337	0.01	0.04	0.798	0.05	0.05	0.287
7	A job where I have a lot of responsibility	0.01	0.05	0.879	0.04	0.05	0.362	0.09	0.05	0.046	-0.07	0.05	0.129	-0.04	0.05	0.369	-0.03	0.05	0.533
8	A job that uses my skills and abilities	-0.01	0.05	0.783	-0.03	0.05	0.502	-0.06	0.05	0.163	0.03	0.05	0.523	0.03	0.05	0.484	0.01	0.05	0.860
9	A job that people regard highly	0.01	0.04	0.735	-0.01	0.04	0.818	-0.03	0.04	0.482	-0.01	0.04	0.785	0.03	0.04	0.452	0.10	0.04	0.025
10	A chance to learn a lot of new things	0.00	0.04	0.920	0.07	0.04	0.100	0.10	0.04	0.025	0.03	0.04	0.484	-0.02	0.04	0.606	-0.02	0.04	0.661
Negative emotions at Time 1		0.05	0.04	0.193	0.05	0.04	0.160	-0.04	0.04	0.302	-0.01	0.04	0.821	-0.03	0.04	0.492	-0.04	0.04	0.321

		**Work values at Time 2**												**Negative emotions at Time 2**		
		Work value 7			Work value 8			Work value 9			Work value 10					
		Coef.	SE	p	Coef.	SE	p	Coef.	SE	p	Coef.	SE	p	Coef.	SE	p
Work values at Time 1																
1	Good pay	0.00	0.04	0.930	-0.01	0.04	0.724	0.05	0.04	0.248	-0.02	0.04	0.564	0.05	0.04	0.268
2	A steady job, with little chance of being laid off	-0.01	0.05	0.752	0.04	0.05	0.436	-0.11	0.04	0.014	-0.05	0.05	0.328	0.07	0.05	0.140
3	Good chances of getting ahead	-0.06	0.05	0.183	0.01	0.05	0.889	-0.06	0.04	0.179	-0.06	0.05	0.205	-0.06	0.05	0.168
4	A chance to be helpful to others or useful to society	0.04	0.05	0.424	-0.02	0.05	0.648	0.07	0.05	0.121	0.08	0.05	0.099	-0.01	0.05	0.770

5	A chance to work with people rather than things	-0.02	0.05	0.731	-0.05	0.05	0.318	-0.08	0.05	0.082	-0.05	0.05	0.265	-0.02	0.05	0.689
6	A chance to make my own decisions	-0.03	0.05	0.484	0.02	0.05	0.659	0.02	0.04	0.733	0.03	0.05	0.535	0.01	0.05	0.827
7	A job where I have a lot of responsibility	0.09	0.05	0.063	-0.04	0.05	0.371	-0.04	0.05	0.389	-0.01	0.05	0.798	0.01	0.05	0.871
8	A job that uses my skills and abilities	0.01	0.05	0.894	0.12	0.05	0.011	0.00	0.05	0.933	0.05	0.05	0.246	-0.01	0.05	0.766
9	A job that people regard highly	0.02	0.04	0.696	0.05	0.04	0.192	0.22	0.04	<0.001	0.06	0.04	0.184	0.03	0.04	0.530
10	A chance to learn a lot of new things	0.02	0.04	0.664	-0.03	0.04	0.435	0.05	0.04	0.269	0.04	0.04	0.412	-0.02	0.04	0.718
Negative emotions at Time 1		0.02	0.04	0.539	-0.01	0.04	0.869	0.00	0.04	0.916	-0.01	0.04	0.791	0.17	0.04	<0.001

Coef.: coefficient; SE: standard error

All coefficients were standardized.

Supplemental Table 14. Associations between work orientations and negative emotions at Time 1 with work orientations and negative emotions at Time 2 in the cross-lagged panel model (N = 684)

	Work orientations at Time 2						Negative emotions at Time 2		
	Extrinsic orientations			Intrinsic orientations					
	Coef.	SE	p	Coef.	SE	p	Coef.	SE	p
Work orientations at Time 1									
Extrinsic orientations	0.08	0.04	0.056	-0.01	0.04	0.864	0.06	0.04	0.143
Intrinsic orientations	0.04	0.04	0.364	0.13	0.04	0.004	-0.04	0.04	0.329
Negative emotions at Time 1	0.03	0.04	0.508	-0.02	0.04	0.696	0.17	0.04	<0.001

Coef.: coefficient; SE: standard error

All coefficients were standardized.

Supplemental Table 15. Associations between work values and self-esteem at Time 1 with work values and self-esteem at Time 2 in the cross-lagged panel model (N = 684)

		**Work values at Time 2**																	
		Work value 1			Work value 2			Work value 3			Work value 4			Work value 5			Work value 6		
		Coef.	SE	p	Coef.	SE	p	Coef.	SE	p	Coef.	SE	p	Coef.	SE	p	Coef.	SE	p
Work values at Time 1																			
1	Good pay	0.18	0.04	<0.001	0.15	0.04	0.001	0.11	0.04	0.011	0.04	0.04	0.348	-0.04	0.04	0.309	-0.01	0.04	0.881
2	A steady job, with little chance of being laid off	0.05	0.04	0.265	0.04	0.05	0.368	-0.03	0.04	0.574	0.01	0.05	0.829	0.04	0.04	0.420	0.02	0.05	0.606
3	Good chances of getting ahead	0.00	0.04	0.972	-0.12	0.05	0.008	-0.02	0.04	0.685	-0.06	0.05	0.191	-0.06	0.04	0.210	0.02	0.05	0.585
4	A chance to be helpful to others or useful to society	0.04	0.05	0.433	0.03	0.05	0.567	0.02	0.05	0.740	0.17	0.05	0.001	0.11	0.05	0.025	0.07	0.05	0.175
5	A chance to work with people rather than things	-0.01	0.05	0.877	-0.02	0.05	0.646	0.00	0.05	0.981	0.01	0.05	0.815	0.15	0.05	0.001	-0.03	0.05	0.561
6	A chance to make my own decisions	-0.03	0.04	0.459	-0.07	0.05	0.153	-0.02	0.04	0.611	0.04	0.05	0.328	0.02	0.04	0.686	0.05	0.05	0.227
7	A job where I have a lot of responsibility	0.00	0.05	0.949	0.04	0.05	0.360	0.08	0.05	0.065	-0.07	0.05	0.118	-0.05	0.04	0.269	-0.04	0.05	0.417
8	A job that uses my skills and abilities	-0.02	0.05	0.660	-0.04	0.05	0.447	-0.07	0.05	0.147	0.03	0.05	0.516	0.03	0.04	0.534	0.01	0.05	0.908
9	A job that people regard highly	0.02	0.04	0.585	0.00	0.04	0.929	-0.03	0.04	0.492	-0.01	0.04	0.793	0.03	0.04	0.402	0.10	0.04	0.021
10	A chance to learn a lot of new things	-0.01	0.04	0.887	0.07	0.04	0.131	0.09	0.04	0.029	0.03	0.04	0.499	-0.03	0.04	0.496	-0.02	0.04	0.576
Self-esteem at Time 1		0.11	0.04	0.007	0.02	0.04	0.540	0.11	0.04	0.007	0.02	0.04	0.630	0.14	0.04	<0.001	0.13	0.04	0.001

		**Work values at Time 2**												**Self-esteem at Time 2**		
		Work value 7			Work value 8			Work value 9			Work value 10					
		Coef.	SE	p	Coef.	SE	p	Coef.	SE	p	Coef.	SE	p	Coef.	SE	p
Work values at Time 1																
1	Good pay	0.01	0.04	0.792	-0.01	0.04	0.842	0.05	0.04	0.200	-0.02	0.04	0.625	0.03	0.04	0.531
2	A steady job, with little chance of being laid off	-0.01	0.05	0.821	0.04	0.04	0.372	-0.11	0.04	0.016	-0.04	0.05	0.376	-0.05	0.04	0.232
3	Good chances of getting ahead	-0.06	0.05	0.169	0.01	0.04	0.910	-0.06	0.04	0.171	-0.06	0.05	0.190	0.11	0.04	0.014
4	A chance to be helpful to others or useful to society	0.04	0.05	0.422	-0.02	0.05	0.629	0.08	0.05	0.118	0.08	0.05	0.104	-0.08	0.05	0.092

5	A chance to work with people rather than things	-0.02	0.05	0.624	-0.06	0.05	0.240	-0.09	0.05	0.059	-0.06	0.05	0.236	0.04	0.05	0.440
6	A chance to make my own decisions	-0.03	0.05	0.537	0.03	0.05	0.577	0.02	0.04	0.673	0.03	0.05	0.491	0.03	0.04	0.525
7	A job where I have a lot of responsibility	0.08	0.05	0.078	-0.05	0.05	0.290	-0.04	0.05	0.337	-0.02	0.05	0.722	0.02	0.04	0.717
8	A job that uses my skills and abilities	0.00	0.05	0.987	0.11	0.05	0.014	-0.01	0.05	0.865	0.05	0.05	0.256	0.04	0.04	0.349
9	A job that people regard highly	0.02	0.04	0.583	0.06	0.04	0.154	0.22	0.04	<0.001	0.06	0.04	0.167	-0.03	0.04	0.534
10	A chance to learn a lot of new things	0.01	0.04	0.809	-0.04	0.04	0.340	0.04	0.04	0.325	0.03	0.04	0.465	0.02	0.04	0.584
Self-esteem at Time 1		0.11	0.04	0.008	0.12	0.04	0.003	0.08	0.04	0.033	0.07	0.04	0.084	0.29	0.04	<0.001

Coef.: coefficient; SE: standard error

All coefficients were standardized.

Supplemental Table 16. Associations between work orientations and self-esteem at Time 1 with work orientations and self-esteem at Time 2 in the cross-lagged panel model (N = 684)

	Work orientations at Time 2						Self-esteem at Time 2		
	Extrinsic orientations			Intrinsic orientations					
	Coef.	SE	p	Coef.	SE	p	Coef.	SE	p
Work orientations at Time 1									
Extrinsic orientations	0.09	0.04	0.026	0.00	0.04	0.909	0.05	0.04	0.234
Intrinsic orientations	0.02	0.04	0.623	0.11	0.04	0.011	0.04	0.04	0.310
Self-esteem at Time 1	0.12	0.04	0.003	0.13	0.04	0.001	0.30	0.04	<0.001

Coef.: coefficient; SE: standard error

All coefficients were standardized.

Supplemental Table 17. Associations between work values and mastery at Time 1 with work values and mastery at Time 2 in the cross-lagged panel model (N = 684)

		**Work values at Time 2**																	
		Work value 1			Work value 2			Work value 3			Work value 4			Work value 5			Work value 6		
		Coef.	SE	p	Coef.	SE	p	Coef.	SE	p	Coef.	SE	p	Coef.	SE	p	Coef.	SE	p
Work values at Time 1																			
1	Good pay	0.18	0.04	<0.001	0.15	0.04	0.001	0.10	0.04	0.012	0.04	0.04	0.384	-0.04	0.04	0.279	-0.01	0.04	0.836
2	A steady job, with little chance of being laid off	0.04	0.04	0.355	0.04	0.05	0.400	-0.03	0.04	0.466	0.01	0.05	0.815	0.03	0.04	0.539	0.02	0.05	0.739
3	Good chances of getting ahead	0.00	0.04	0.972	-0.12	0.05	0.008	-0.02	0.04	0.640	-0.06	0.05	0.200	-0.06	0.04	0.190	0.02	0.05	0.633
4	A chance to be helpful to others or useful to society	0.04	0.05	0.447	0.03	0.05	0.571	0.01	0.05	0.759	0.17	0.05	0.001	0.11	0.05	0.026	0.07	0.05	0.176
5	A chance to work with people rather than things	0.00	0.05	0.968	-0.02	0.05	0.680	0.01	0.05	0.852	0.01	0.05	0.809	0.16	0.05	0.001	-0.02	0.05	0.708
6	A chance to make my own decisions	-0.04	0.04	0.368	-0.07	0.05	0.140	-0.03	0.05	0.503	0.05	0.05	0.311	0.01	0.04	0.816	0.05	0.05	0.294
7	A job where I have a lot of responsibility	0.01	0.05	0.777	0.05	0.05	0.331	0.09	0.05	0.040	-0.07	0.05	0.116	-0.04	0.05	0.397	-0.03	0.05	0.565
8	A job that uses my skills and abilities	-0.02	0.05	0.601	-0.04	0.05	0.424	-0.07	0.05	0.127	0.03	0.05	0.473	0.03	0.05	0.565	0.00	0.05	0.950
9	A job that people regard highly	0.02	0.04	0.549	0.00	0.04	0.953	-0.03	0.04	0.529	-0.01	0.04	0.737	0.03	0.04	0.402	0.10	0.04	0.021
10	A chance to learn a lot of new things	-0.01	0.04	0.893	0.07	0.04	0.134	0.09	0.04	0.030	0.03	0.04	0.450	-0.03	0.04	0.545	-0.02	0.04	0.613
Mastery at Time 1		0.07	0.04	0.065	0.03	0.04	0.515	0.07	0.04	0.062	-0.03	0.04	0.499	0.06	0.04	0.107	0.06	0.04	0.110

		**Work values at Time 2**												**Mastery at Time 2**		
		Work value 7			Work value 8			Work value 9			Work value 10					
		Coef.	SE	p	Coef.	SE	p	Coef.	SE	p	Coef.	SE	p	Coef.	SE	p
Work values at Time 1																
1	Good pay	0.01	0.04	0.870	-0.01	0.04	0.860	0.05	0.04	0.242	-0.02	0.04	0.582	0.03	0.04	0.490
2	A steady job, with little chance of being laid off	-0.02	0.05	0.737	0.03	0.04	0.501	-0.11	0.04	0.013	-0.04	0.05	0.337	0.02	0.04	0.619
3	Good chances of getting ahead	-0.06	0.05	0.168	0.00	0.04	0.992	-0.06	0.04	0.175	-0.06	0.05	0.186	0.00	0.04	0.925
4	A chance to be helpful to others or useful to society	0.04	0.05	0.409	-0.02	0.05	0.630	0.08	0.05	0.116	0.08	0.05	0.099	-0.04	0.05	0.378

5	A chance to work with people rather than things	-0.02	0.05	0.741	-0.05	0.05	0.330	-0.08	0.05	0.081	-0.05	0.05	0.271	0.06	0.05	0.218
6	A chance to make my own decisions	-0.03	0.05	0.482	0.02	0.05	0.700	0.02	0.04	0.725	0.03	0.05	0.524	0.00	0.04	0.929
7	A job where I have a lot of responsibility	0.09	0.05	0.058	-0.04	0.05	0.429	-0.04	0.05	0.393	-0.01	0.05	0.794	0.07	0.05	0.154
8	A job that uses my skills and abilities	0.00	0.05	0.960	0.11	0.05	0.022	0.00	0.05	0.920	0.05	0.05	0.259	0.03	0.05	0.460
9	A job that people regard highly	0.02	0.04	0.613	0.06	0.04	0.127	0.22	0.04	<0.001	0.06	0.04	0.174	-0.01	0.04	0.792
10	A chance to learn a lot of new things	0.01	0.04	0.740	-0.04	0.04	0.324	0.05	0.04	0.279	0.03	0.04	0.431	-0.04	0.04	0.322
Mastery at Time 1		0.02	0.04	0.575	0.10	0.04	0.010	0.00	0.04	0.924	0.02	0.04	0.621	0.23	0.04	<0.001

Coef.: coefficient; SE: standard error

All coefficients were standardized.

Supplemental Table 18. Associations between work orientations and mastery at Time 1 with work orientations and mastery at Time 2 in the cross-lagged panel model (N = 684)

	Work orientations at Time 2						Mastery at Time 2		
	Extrinsic orientations			Intrinsic orientations					
	Coef.	SE	p	Coef.	SE	p	Coef.	SE	p
Work orientations at Time 1									
Extrinsic orientations	0.08	0.04	0.049	0.00	0.04	0.913	0.04	0.04	0.363
Intrinsic orientations	0.03	0.04	0.443	0.12	0.04	0.005	0.04	0.04	0.315
Mastery at Time 1	0.05	0.04	0.237	0.05	0.04	0.159	0.22	0.04	<0.001

Coef.: coefficient; SE: standard error

All coefficients were standardized.

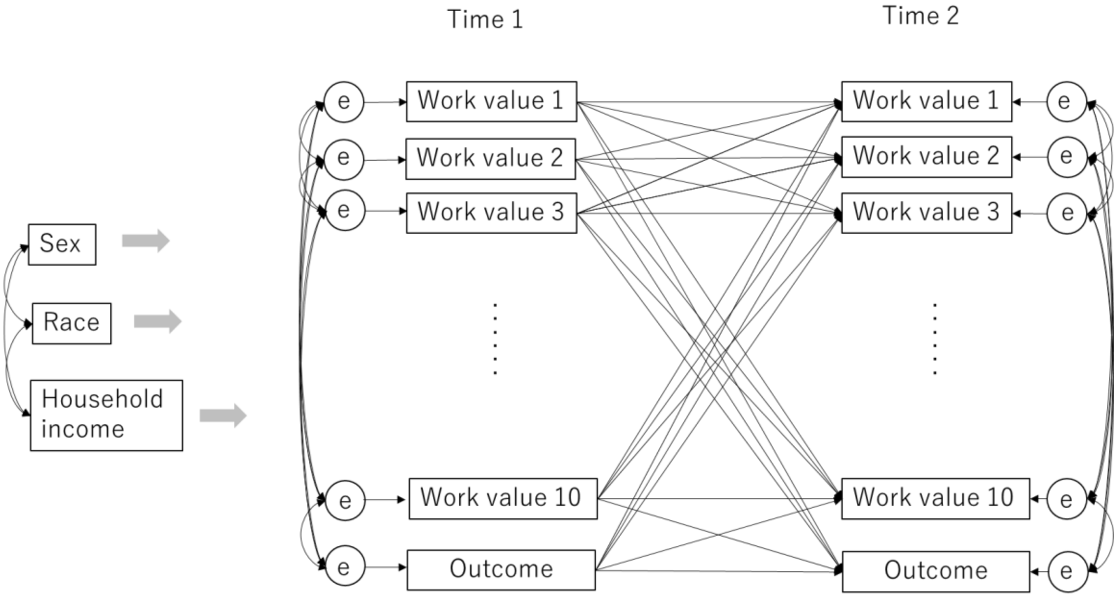

Supplemental Figure 1. Cross-lagged panel model estimating the effects of work values and an outcome at Time 1 on those at Time 2. Direct effects of socio-demographic variables (sex, race, and household income) on all 10 work values and an outcome at Time 1 and Time 2 were assumed but omitted from the above figure for simplicity.

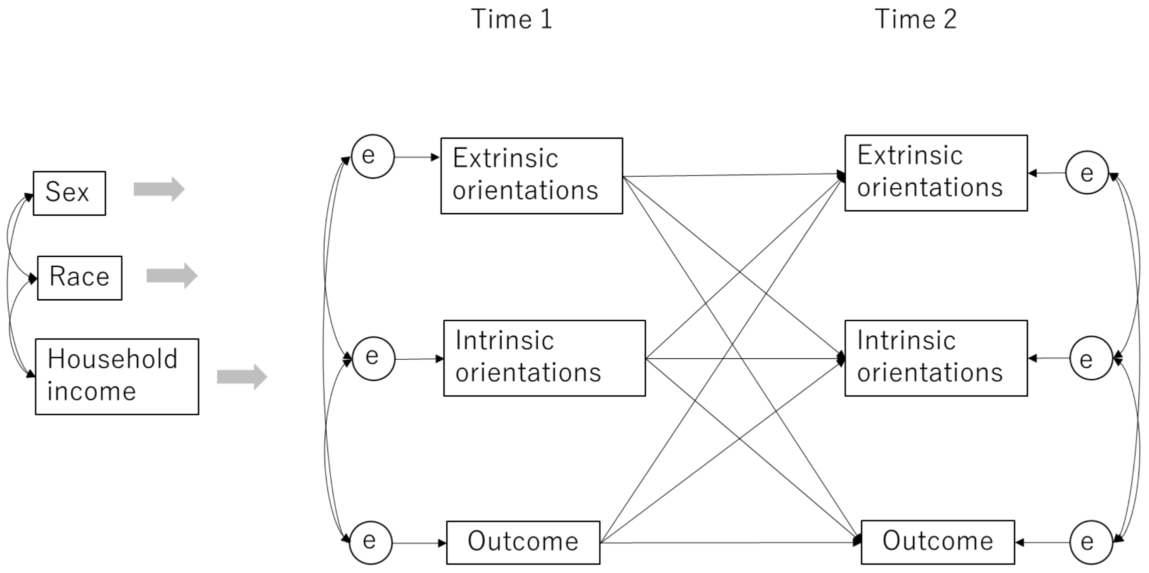

Supplemental Figure 2. Cross-lagged panel model estimating the effects of work orientations and an outcome at Time 1 on those at Time 2. Direct effects of socio-demographic variables (sex, race, and household income) on two work orientations and an outcome at Time 1 and Time 2 were assumed but omitted from the above figure for simplicity.
